# Coupled Protein Diffusion and Folding in the Cell

**DOI:** 10.1371/journal.pone.0113040

**Published:** 2014-12-01

**Authors:** Minghao Guo, Hannah Gelman, Martin Gruebele

**Affiliations:** 1 Department of Physics, University of Illinois, Urbana, IL, United States of America; 2 Department of Chemistry and Center for Biophysics and Computational Biology, University of Illinois, Urbana, IL, United States of America; Weizmann Institute of Science, Israel

## Abstract

When a protein unfolds in the cell, its diffusion coefficient is affected by its increased hydrodynamic radius and by interactions of exposed hydrophobic residues with the cytoplasmic matrix, including chaperones. We characterize protein diffusion by photobleaching whole cells at a single point, and imaging the concentration change of fluorescent-labeled protein throughout the cell as a function of time. As a folded reference protein we use green fluorescent protein. The resulting region-dependent anomalous diffusion is well characterized by 2-D or 3-D diffusion equations coupled to a clustering algorithm that accounts for position-dependent diffusion. Then we study diffusion of a destabilized mutant of the enzyme phosphoglycerate kinase (PGK) and of its stable control inside the cell. Unlike the green fluorescent protein control's diffusion coefficient, PGK's diffusion coefficient is a non-monotonic function of temperature, signaling ‘sticking’ of the protein in the cytosol as it begins to unfold. The temperature-dependent increase and subsequent decrease of the PGK diffusion coefficient in the cytosol is greater than a simple size-scaling model suggests. Chaperone binding of the unfolding protein inside the cell is one plausible candidate for even slower diffusion of PGK, and we test the plausibility of this hypothesis experimentally, although we do not rule out other candidates.

## Introduction

Macromolecular crowding in the cell modulates protein structure and stability, as well as protein diffusion and transport [1], [2]. The crowded environment of the cell limits protein diffusion and gives rise to anomalous diffusion on long time scales [3], [4], as well as position-dependent diffusion [5], [6]. Anomalous diffusion in living cells has been studied extensively by fluorescence recovery after photobleaching (FRAP) [5], [7], [8] and by fluorescence correlation spectroscopy (FCS) [4], [9], [10]. However, both methods focus on local diffusion, providing little information about the global cellular environment. Fluorescence loss in photobleaching (FLIP), while it gives up precise details about short distance behavior, has the potential to provide a larger scale view of diffusion [11].

So far, none of these techniques have been used to look at the coupling of protein folding and diffusion inside living cells. After initial translation, proteins of typical stability unfold and refold many times in the cell during their lifecycle [12]. Other proteins (sometimes referred to as “intrinsically disordered proteins”) diffuse mostly while unfolded, and fold only upon binding to a signaling partner [13]. One expects that protein diffusion in the cell slows down when a protein unfolds, either due to its increased hydrodynamic radius and crowding, or because the newly exposed hydrophobic residues are ‘sticky’ when interacting with other macromolecules in the cytoplasm [14]. A regime where unfolded polymer chains could diffuse faster than spheroid polymers among highly crowding obstacles is also possible in principle [15], [16], but it seems less likely at the moderate (300–400 mg/mL) crowding conditions inside cells. To complicate matters *in vivo* even more, hydrodynamic effects (e.g. the dragging of solvent molecules by macromolecules) could contribute to anomalous diffusion, and to the value of effective diffusion coefficients [17], and hydrodynamic effects could be significantly different for folded *vs*. unfolded proteins inside cells. For all of these reasons, more experimental data is needed on how biomolecular shape in general, and folding/unfolding specifically, affects diffusion in cells.

**Figure 1:**
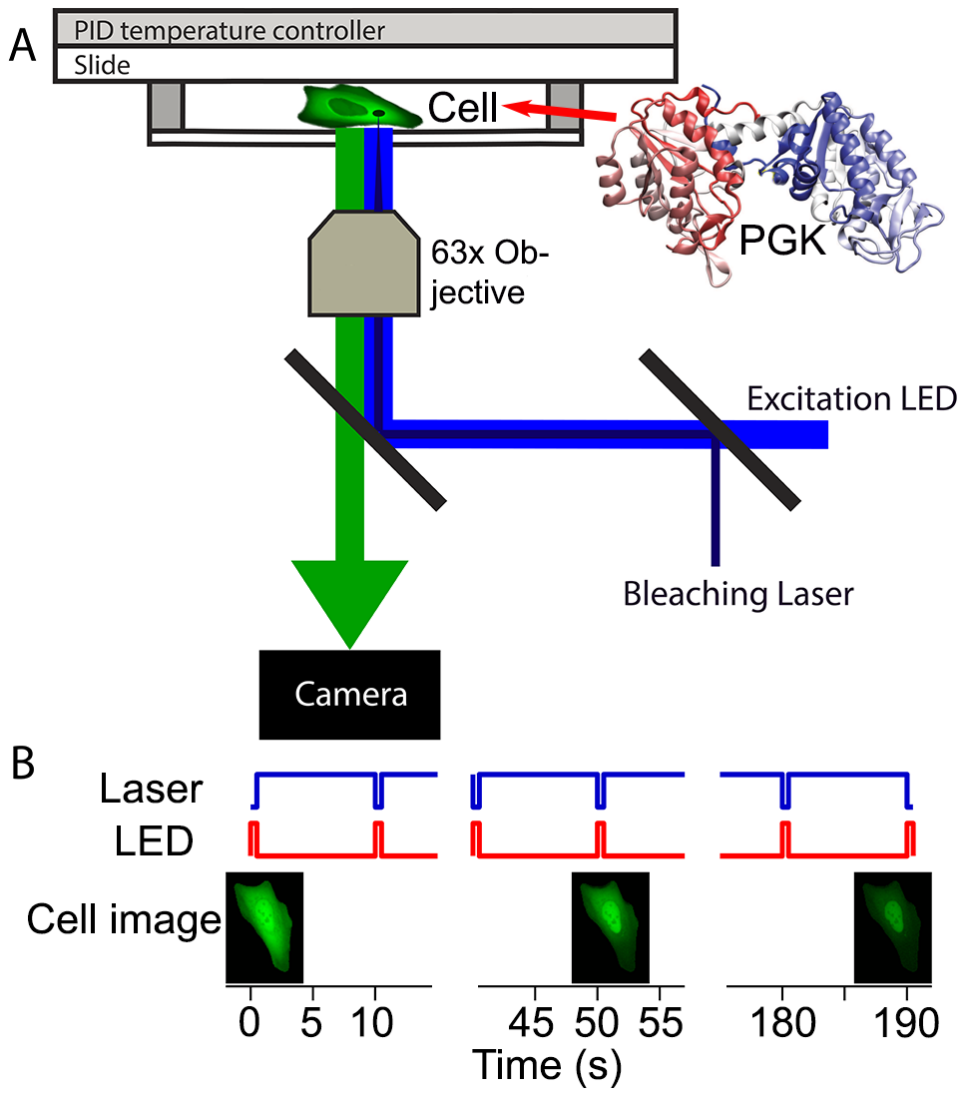
The instrument for FLIP measurements. (A) The imaging LED and bleaching laser are combined to excite GFP or GFP-labeled proteins in the cell (ribbon structure of PGK at the top right). The LED illuminates the whole cell evenly for imaging. The laser is focused to a small intense spot (see Methods) to locally photobleach the fluorescent protein. (B) The LED and laser are controlled to turn on alternately every 10 seconds. Snapshots are taken with only the LED on to record the progress of the fluorescence intensity decay in the cell without saturating the camera.

We begin by characterizing cytoplasmic protein diffusion in U2OS cells at 22°C, using FRAP as a benchmark. In FRAP, a small spot in the cell is bleached, and the recovery of fluorescent proteins diffusing into the depleted spot is monitored. We then use FLIP with GFP as a model protein to characterize position-dependent anomalous diffusion in the cell. In the FLIP experiment, a focused laser also photobleaches the fluorescent protein at a small spot in the cell (Figure 1). Proteins everywhere in the cell are imaged as they replenish the bleached spot by diffusion. The imaged fluorescence intensity gradually drops throughout the cytoplasm, and the kinetics of bleaching can be analyzed to study the global diffusion behavior. We use 2-D and 3-D diffusion simulations to model our global diffusion data from FLIP. We develop a clustering algorithm to divide the cell into several partitions based on local diffusion behavior, spatially resolving diffusion in the cell. This method can be used to fully utilize the global information measured by FLIP experiments to study the spatial heterogeneity of diffusion and anomalous diffusion in the cell.

Finally, we use the temperature-dependence of a protein's diffusion coefficient as a handle to detect protein unfolding in the crowded cytosol of U2OS cells. We study variants of the enzyme phosphoglycerate kinase (PGK) because their thermal stability and kinetics have been thoroughly studied *in vitro*, in crowders, and in live cells by the FREI technique [18], [19]. A ribbon structure of PGK is shown in Figure 1. We measure the diffusion of a low temperature-unfolding mutant of PGK labeled with GFP only (ltPGK-GFP), and labeled with both GFP and mCherry for FRET detection of folding (ltPGK-FRET). As controls, we measure the diffusion of GFP as a function of temperature (melting temperature>65°C), and of htPGK-GFP, a higher temperature-unfolding mutant of PGK labeled with GFP; neither of these proteins unfolds over the temperature range of our experiment. A simple size-dependent model qualitatively explains how protein size and stability affect translational diffusion in U2OS cells, but underestimates the experimentally observed changes. The observed large speedup and slowdown of diffusion when the temperature is increased may be caused by a trade-off between increased protein and cytoplasmic matrix flexibility on the one hand, and increased ‘stickiness’ of the unfolded protein due to exposed hydrophobic residues on the other hand.

Natural candidates for interaction with PGK's exposed hydrophobic residues are the cell's pool of chaperone proteins, which are thought to bind exposed hydrophobic residues to prevent protein misfolding and aggregation [20], [21]. We show with an in-cell binding assay that, as it unfolds, ltPGK-GFP binds to fluorescently-labeled Hsp70, a human chaperone protein of which PGK is a client [22]. Thus chaperone binding is one plausible mechanism for the enhanced ‘stickiness’ and greater-than-expected reduced diffusion coefficient of unfolded PGK in the cytosol.

## Materials and Methods

A brief summary of protein expression, instrumentation, measurements and analysis is given here. Additional details and figures illustrating the analysis are shown in File S1.

### Samples

The U2OS human bone cancer cell line was used for the live cell diffusion studies. The cell line was directly purchased from ATCC, catalog number HTB-96. Some experiments were conducted in the same cell line provided by Prof. Supriya Prasanth. U2OS cells were transfected using lipofectamine with a plasmid encoding the protein of interest for diffusion and Hsp binding experiments. All diffusion experiments were conducted using an N-terminal GFP tag as both the target of photobleaching and the probe for imaging. The “lt”  =  “low melting temperature” mutant ltPGK contains 3 mutations from the wild-type sequence: Y122W/W308F/W333F. ltPGK-FRET in addition has an mCherry at the C-terminus. Its unfolding can be detected by FRET, yielding unfolded population *u*(*T*) ≈ *K_eq_*/(1+*K_eq_*), where *K_eq_*  =  exp[-*ΔG*/*RT*]. *ΔG*  =  *δg*_T_(*T*−*T*_m_) is the two-state unfolding free energy, and the parameters *δg*_T_ and *T*_m_ were fitted as in refs. [23], [24]. *u*(*T*) is shifted *ca.* 2.5°C more stable in-cell than *in vitro* [25]. We measured the stability of the lt mutant with a GFP tag at the N-terminus (ltPGK-GFP) by tryptophan fluorescence *in vitro* (Figure S1 in File S1) and corrected it by the same 2.5°C difference as measured directly for ltPGK-FRET. The stable mutant htPGK contains Y122W/P111T mutations from wild-type.

### Live cell FLIP and FRAP

Cellular diffusion was measured on an epi-fluorescence microscope. A 440 nm blue laser (5 mW, spot size of 4 µm in diameter) bleached the cell, while a 470 nm excitation LED imaged the protein distribution in the cell. Cells with different expression levels (protein concentrations) were measured, showing no correlation of diffusion with concentration (Figure S2 in File S1). In the FLIP measurements, the sample was illuminated as shown in Figure 1 The temperature of the sample slide was controlled by a resistive heater and PID controller within 0.1°C stability [25].

FRAP measurements were performed with the same setup and cell line. Bleaching at the laser spot was carried out for 100 ms. Immediately after bleaching, a video of fluorescence recovery around the bleaching spot was recorded under LED illumination for 10 seconds at 1000 frames per second (fps). A snapshot taken prior to the application of the bleaching laser pulse was used for reference. The relative intensity change compared to the initial values was fitted to a Gaussian to determine the diffusion coefficient [26].

### Simulation of protein diffusion in cells

In the 2-D simulations of diffusion and photobleaching, molecules are allowed to diffuse in a grid area corresponding to the shape of the imaged cell. Grid size is *Δx* = 0.85 µm, corresponding to the pixel resolution of the recorded image. For 2-D simulations, the experimental data is normalized by the initial pixel intensities, assuming initial concentration of molecules in the model is uniform. The nucleus is excluded from the diffusion accessible region.

The simulated laser beam is cylindrically shaped, with Gaussian radius *σ* = 2.5 µm. The number of protein molecules photobleached per unit time is proportional to the photobleaching rate *B*(*x*,*t*)  = *B*.A(*t*).exp[-(*x* ^2^+*y* ^2^)/*σ* ^2^] and molecule concentration *n*(*x*,*t*). *A*(*t*)  = 0 (bleaching laser off) or 1 (bleaching laser on) as plotted in Figure 1B. The diffusion of protein molecules was modeled by solving Fick's law with a concentration sink, representing the bleaching of labeled molecules:



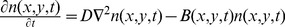
(1)


Analogous equations were solved for 3-D diffusion, anomalous diffusion [27] [28], and for multi-domain diffusion, as detailed in File S1.

### Clustering and partitioning of the cell

The fluorescence intensity decay of each pixel in the experiment is first compared with the homogeneous simulation fit (*D* =  constant). Pixels with similar dynamics are grouped together based on the residual between the experimental and simulated intensities by using the k-means algorithm [29] iteratively until a few smooth domains were obtained (see File S1). With our signal-to-noise ratio, we found that 3–4 domains accounted for the data within measurement uncertainty. Different values of *D*(*x*,*y*) and *α*(*x*,*y*) are used for each domain, and smoothed using a 10-pixel box filter to avoid sudden changes in *D* at the domain boundaries.

### PGK-Hsp70 binding in the cell

A fluorescent hsp70 fusion protein was created by cloning the sequence for the human, cytoplasmic, heat-inducible hsp70 (hsp72) [30] with a C-terminal mCherry tag into the pDream 2.1/MCS vector (Genscript Corp., Piscataway, NY). The construct also includes N-terminal FLAG and hexahistidine tags. C-terminal fluorescent protein fusions of Hsp-70 have been shown to maintain chaperone activity and to co-express and co-localize with native Hsp70 under heat shock conditions [31]. Red and green fluorescence from mCherry and GFP was separated into two channels by a dichroic mirror and imaged side-by-side on a CMOS camera sensor. Cells were imaged under steady illumination at temperatures from 20°C–45°C to create a temperature titration curve.

Binding between hsp70-mCherry and GFP-PGK was detected *via* FRET from GFP to mCherry. FRET was quantified as in [24]: the ratio of average donor to acceptor fluorescence (*D*/*A*) was calculated for each cell at each temperature. As the interaction between the Hsp70-mCherry and GFP-PGK increases, the FRET efficiency between the two fluorescent tags increases and *D*/*A* decreases. The change in the ratio *D*/*A* with respect to temperature was fitted by a two-state model identical to the one described in the FLIP and FRAP Methods section.

## Results

### FRAP *vs.* FLIP experiments

FRAP has been applied widely to measure diffusion coefficients of fluorescently-labeled biomolecules in cells. However, it measures only one point of interest at a time. Such a localized result serves as a useful comparison with global FLIP measurements, where the entire cell is imaged during fluorescence depletion. We measured the GFP diffusion coefficient at 22°C at several locations in the cytoplasm of five U2OS cells by FRAP (see Methods). FRAP measurements yielded an average *D* = 21 µm^2^/sec at 22°C, with a range of 5–47 µm^2^/sec and a standard deviation of ±17 µm^2^/sec.

**Figure 2:**
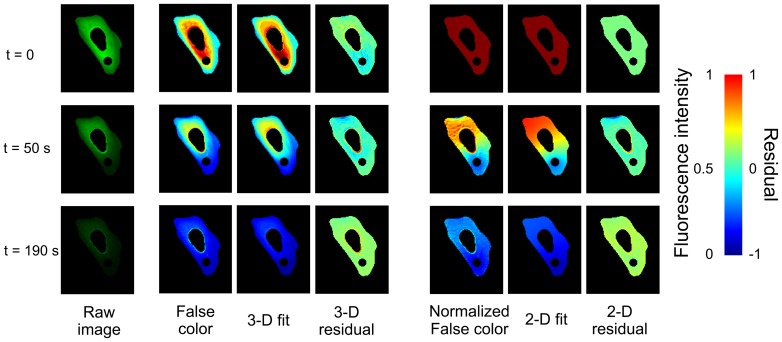
Snapshots of the GFP fluorescence intensity at three of the 19 time steps sampled during bleaching in U2OS cells. Left column: FLIP data in true color; the small bleaching spot and nucleus are excluded from analysis. Middle three columns: FLIP data in false color for better contrast (scale bar at right), 3-D diffusion model fit, and fit residual. Rightmost three columns: Same FLIP data in false color normalized to the fluorescence intensity distribution at *t* = 0, 2-D diffusion model fit, and fit residual.

Figure 2 illustrates the FLIP measurement for one of four cells measured at 22°C (see Movie S1 for a measurement example). Utilizing the models discussed below, whole-cell FLIP measurements yielded an average *D* = 17.1 µm^2^/sec at 22°C with a range of 11–28 µm^2^/sec and a cell-to-cell standard deviation of ±6.5 µm^2^/sec. Thus the average FRAP and FLIP diffusion coefficients lie within the cell-to-cell standard deviation, which reflects cell variations as well as measurement error. Based on *in vitro* measurements of diffusion in GFP solutions, most of the standard deviation is from cell-to-cell (FLIP) and point-to-point (FRAP) variations, not measurement error of the instrument.

The smaller ±6.5 µm^2^/sec standard deviation of the whole-cell FLIP measurement *vs.* ±17 µm^2^/sec from FRAP indicates that roughly a third of the variation of the observed diffusion coefficients comes from cell-to-cell variation, and the other two thirds comes from the heterogeneous environment within individual cells. As we shall see below, FLIP can be used to systematically sample regional diffusion environments and to image these environments within a single cell.

### Modeling of protein diffusion in cells detected by FLIP

To determine the diffusion coefficient *D* from the experimental FLIP data in Figure 2, we solved 2-D and 3-D models for normal diffusion, i.e. where the mean squared displacement is proportional to time (<*x* ^2^> = 6*Dt*, see Methods). Due to the complex and varied shapes of the cells, we used a numerical simulation to analyze the photobleaching and diffusion behavior in each individual cell. The simulation solves the coupled differential equations describing labeled protein molecules diffusing throughout the cell and photobleaching as they pass through the laser spot. We iteratively adjusted two parameters to fit the simulation to the FLIP experimental data: the diffusion coefficient *D* averaged over the cell (excluding nucleus and bleaching spot), and the bleaching rate *B* of molecules inside the laser spot.

**Figure 3:**
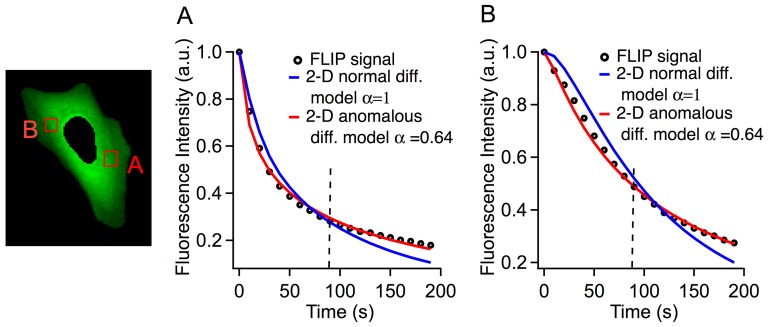
Comparison of experimental data with 2-D normal and anomalous diffusion models at the two locations in the cell (A) and (B). The experimental fluorescence intensity (black circles) initially decays faster, and subsequently slower than the best normal diffusion model fits (blue curves). Only anomalous diffusion with α<1 (red curves) correctly simulates the observed data. The vertical dashed lines at 85 s indicate where the short time (horizontal axis in Figure 4) and long time (vertical axis in Figure 4) residuals were calculated. The normal diffusion model tends to have a negative residual at short time and a positive residual at long time, whereas the anomalous diffusion model has much smaller residuals.

**Figure 4:**
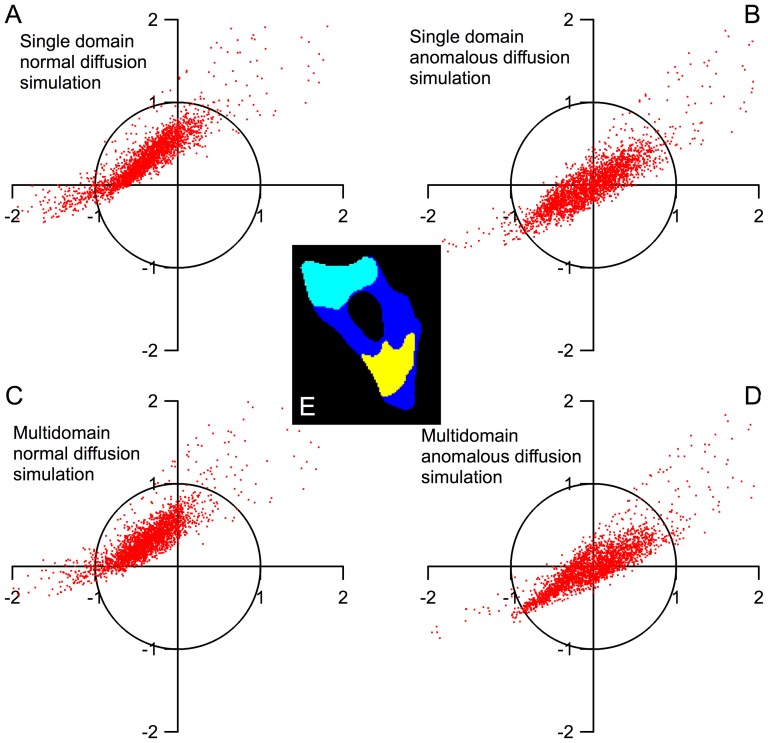
The distribution of the residuals of all the pixels in the cell. (A–E) Single and multi-domain models of normal and anomalous diffusion simulations. The pixels represent the residual of a single pixel in the cell at *t*≤90 s (x-axis) and *t*≥100 s (y-axis). Normal diffusion simulations (A,C) have systematic deviation from experiment results, visualized by an offset in the residual graph. In the anomalous diffusion simulations (B,D), the multi-domain model results in smaller overall residuals than the single domain model. (E) The illustration of the three domains in the cell calculated by the *k*-means clustering algorithm.

**Figure 5:**
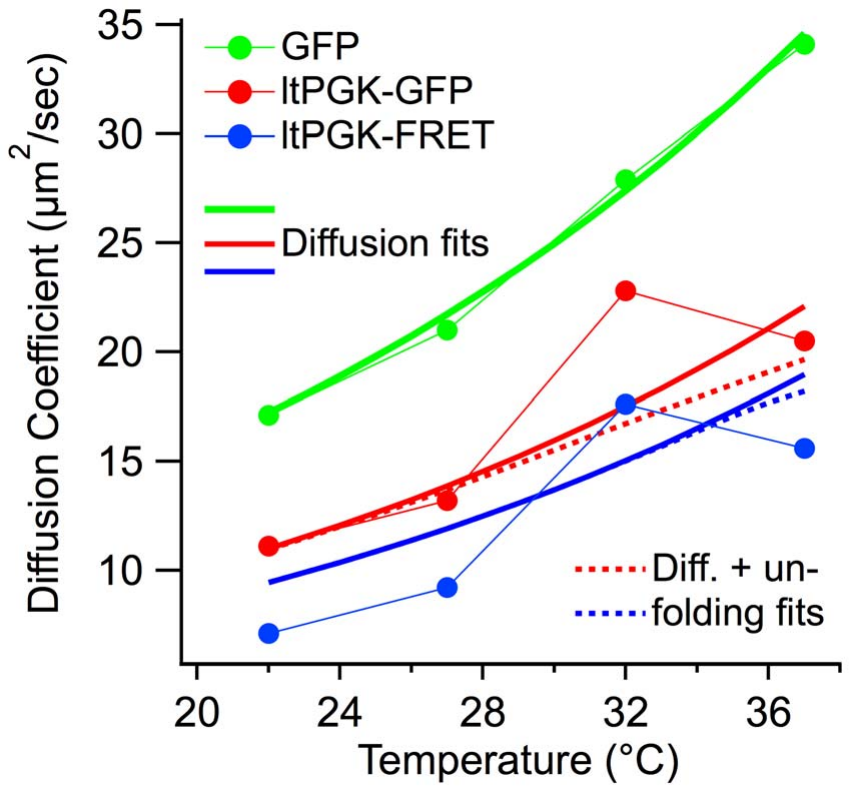
Comparison of the diffusion coefficients of the GFP, ltPGK-GFP and ltPGK-FRET measured from 22°C to 37°C. All proteins diffuse faster at higher temperatures while folded. The “lt” proteins show accelerated diffusion followed by a turnaround at *T* near *T*_m_. Global model fits are to equation 1 (solid thick lines) and equation 2 (dotted thick lines).

The 2-D model assumes that the cells adhered to microscope slides are flat enough to be approximated by slabs of constant thickness, ignoring any depth variation across the cell. To validate this assumption, we also fitted the experimental data to a more time-consuming 3-D simulation in which cell thickness is modeled as being proportional to the fluorescence intensity at each pixel before bleaching begins. The diffusion coefficients from the two models are very similar, and the residuals of both models in Figure 2 are equally small. The 3-D model simulations also show the same anomalous diffusion discussed below. Our estimation method of the cell thickness for 3-D simulations has limited accuracy. The 2-D model is simpler, faster and equally accurate for in-cell diffusion monitored by FLIP because FLIP integrates over the vertical height of the cell in each image pixel. Therefore all the data analysis reported in Tables 1 and 2, and in Figures 3-5 is derived from the 2-D model.

**Table 1:** 2-D model fits for GFP diffusion inside the cell shown in Figures 2-4.

Model type	Fitted diffusion coefficient *D* (µm^2^/sec^α^)	Fitted anomalous factor *α*	Normalized mean squared deviation
A. Single domain, normal diffusion	34.2	1	1
B. Single domain, anomalous diffusion	222.8	0.64	0.87
C. Multi domain, normal diffusion	34.0 (blue); 204.2 (cyan); 16.5 (yellow)	1*	0.89†
D. Multi domain, anomalous diffusion	293.7 (blue); 2875 (cyan); 58.6 (yellow)	0.79 (blue); 0.55 (cyan); 0.90 (yellow)	0.73†

The color names in fits C and D refer to the regions of the cell in Figure 4E.

* Anomalous factor α fixed at 1 in all regions for multi domain, normal diffusion simulations.

† Mean squared deviation calculated across all regions in multi domain simulations.

**Table 2:** Mass and melting temperature of the main proteins in this study, as well as number of cells *N* measured by FLIP and standard deviation *σ* (in µm^2^/s) of the average measured diffusion coefficients (shown in Figure 5) among the *N* cells.

Molecular Mass	GFP	ltPGK-GFP	ltPGK-FRET
	28 kDa	73 kDa	101 kDa
*T*_m_ *	65±1°C	37±1°C	39±1°C
22°C	*N* = 4, *σ* = 6.5	*N* = 3, *σ* = 1.8	*N* = 2, *σ* = 2.0
27°C	*N* = 4, *σ* = 7.7	*N* = 4, *σ* = 4.1	*N* = 7, *σ* = 2.3
32°C	*N* = 3, *σ* = 6.1	*N* = 4, *σ* = 8.2	*N* = 4, *σ* = 5.2
37°C	*N* = 5, *σ* = 9.2	*N* = 3, *σ* = 2.0	*N* = 5, *σ* = 5.4

*In vitro; the in-cell melting temperature of the ltPGK-FRET mutant is *ca.* 2.5°C higher, and unknown for the ltPGK-GFP mutant.

### Normal and anomalous diffusion

Although the normal diffusion models in Figure 2 describe the overall diffusion in the entire cell fairly well, they are by no means exact at individual spots within the cell. Figure 3 compares the fluorescence intensity decay due to photobleaching at two locations in a single cell at 22°C. Location B is further from the laser bleaching spot than A, so the initial fluorescence decay at B is slower. The 2-D normal diffusion model is able to qualitatively match the kinetics of photobleaching at the two points (blue fits). However, the fits at both points show that the normal diffusion fit underestimates the bleaching rate at short times and overestimates the bleaching rate at longer times. We quantify these deviations by assigning the average fit residuals at short (10–90 s) and long (100–190 s) times to be the components of a 2-D vector (*x* and *y*, respectively). This division (see line in Figure S3 in File S1 and dashed lines in Figure 3BC) was chosen because it is the point at which the normal diffusion fit switches from a negative to a positive residual. Each image pixel in the cell has an associated (x,y) vector and the distribution of fit deviations can be plotted on a two dimensional graph (see Figure S3 in File S1 and Figure 4AB). Systematic deviations of the fit at short and long times can be visualized as a displacement of this distribution from the origin of the graph (see Figure S4 in File S1). The short- and long- time deviations in the normal diffusion fits indicate that the diffusion measured by FLIP is anomalous diffusion where <*x^2^*>∼*t^α^*, *α*<1. We implemented a 2-D anomalous diffusion model (see Methods) and found that *α* = 0.64 better fit the GFP data at both points in the cell shown in Figure 2 and in Figure 3BC (red fits). ltPGK-GFP diffusion at 22°C in a different cell was fitted to the same model, yielding *α* = 0.59, in close agreement. These findings of anomalous diffusion are an indication that molecular crowding in the cytosol reduces the ability of proteins to diffuse over long distances.

### Spatial dependence of diffusion

The anomalous diffusion simulation improves on the normal diffusion model, but still leaves positive or negative residuals at different locations within the cell. As we saw in the FRAP experiments, diffusion coefficients are spatially heterogeneous within the cell. This is evident in the FLIP data as well. For example, the anomalous diffusion model with a single diffusion coefficient of the cell in Figure 3 slightly overestimates the bleaching rate at long times at point A (Figure 3B), while it overestimates the bleaching rate at short times at point B (Figure 3C). To incorporate spatial heterogeneity in the FLIP analysis, we divide cells into *N* domains, extend the 2-D model to include both α≠1 and *N* domains with different values of *D*, and employ a clustering algorithm to partition the cell into domains with a similar diffusion coefficient (see Methods and Figure S5 in File S1). Of course such a coarse-grained model can only account for longer-range variations of *D*. For the cell shown in Figure 4E, we fitted *N = 3* regions as the optimal compromise between enhancing the accuracy of the fit and limiting the number of fitting parameters.

Figure 4 compares the resulting 2-D model fits with the homogeneous normal diffusion model (α = 1, *N* = 1 =  single domain). The residuals from each model fit are visualized as described above and plotted in Figure 4A–D. Each point in Figure 4A–D represents the residual at a single pixel of the cell in Figure 4E. Table 1 lists the fitted values of diffusion coefficients and *α* values for models A–D, as well as the mean squared deviation of each model. Diffusion coefficients change up to 7-fold in different areas of the cell. This result is consistent with the FRAP measurement discussed previously, where *D* varied from 5–47 µm^2^/sec between measurements taken at individual points in the cell. Table 1 also shows that *α* varies from 0.55 to 0.90 in different areas in the cell, and that anomalous diffusion and position-dependent diffusion contribute about equally to the improved fit compared to the *α* = 1, *N* = 1 model. The remaining variation (“cloud of points”) in Figure 4 comes from measurement error and short-range variations of *D* and *α*, which we cannot disentangle at present.

### Coupled diffusion and folding

Having characterized protein diffusion in the U2OS cell mainly with GFP, we turn to folding-dependent diffusion as a function of temperature. As a control, we measured temperature-dependent diffusion of GFP, whose melting point lies well above our temperature window (Table 2). Next, we investigated temperature-dependent diffusion of ltPGK-GFP and ltPGK-FRET, both of which have in-cell melting temperatures ≤42°C. Above ≈40°C, cells were not healthy for the long (250 s) FLIP measurement, as evidenced by observation of cell morphology changes [32]. The measured diffusion coefficients *D* are calculated using the single-domain, normal diffusion model to provide a global average value. They are plotted in Figure 5 from 22 to 37°C (solid circles). Table 2 summarizes the protein masses, melting temperatures, number of cells measured and cell-to-cell standard deviation of *D* for each temperature/protein combination measured. Within the temperature range from 22°C to 28°C, the temperature- and size-dependence of *D* is as expected: ltPGK-FRET diffuses the slowest due to its large size; the GFP control has the smallest mass and the largest diffusion coefficient; and diffusion speeds up at higher temperature. Above 28°C, diffusion continues to speed up with temperature for the GFP control, while the diffusion coefficient of both ltPGK-GFP and ltPGK-FRET shows a marked decrease at higher temperatures.

To better understand the temperature dependent changes in *D*, we employed a model that relates *D* of both globular proteins and flexible polymers in aqueous solution to the temperature and molecular mass *M* by: 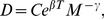
(2) where *C* is a constant, *T* is in Kelvin, and *β* accounts for the temperature dependence of *D* over a small temperature window. *γ* is a shape factor that describes the globularity of the protein. Measurements in the literature have shown that it lies typically between 0.33 (globule-shaped protein) and 0.56 (elongated protein) [33]. Both PGK and the fluorescent proteins lie between these limits (see Figure 1), so we expect an intermediate value. Indeed, *β* = 0.04 and *γ*  = 0.42 (thick solid fitted curves in Figure 5) agree with the experimental diffusion coefficients in Figure 5 fairly well from 22 to 27°C, although PGK-FRET diffuses even more slowly than predicted. The complex structure of PGK-FRET – three globular proteins tethered together – may be responsible for this slower diffusion. The tethered protein may have a larger Stokes radius than a single compact globular protein of the same mass, which is assumed in eq. (2). (Note: the Stokes radius is the radius of a perfectly spherical particle with the same diffusion coefficient as the protein.)

Above 27°C, the same model continues to nicely fit the data for GFP, but it fails to account for the experimentally observed turnaround of *D* of the low-melting PGK variants. We assign this turnaround to unfolding of ltPGK, which begins at 35°C; unfolding increases the Stokes radius of the proteins and may also make them more ‘sticky’ because exposed hydrophobic residues can interact with cytoplasmic constituents. To see if we could fit the higher temperature data, we extended the model of eq. (2) by adding a scaling coefficient *s*>1 for the unfolded protein:
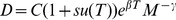
(3)


where *u*(T) is the unfolded protein fraction as a function of temperature, *ca.* 0.2 for ltPGK-FRET, and *ca*. 0.5 for ltPGK-GFP at 37°C (see Methods). The results of the model fit are shown as dotted lines in Figure 5. Although the extended model shows a trend towards smaller diffusion coefficients at higher temperature, it does not account quantitatively for the increase and subsequent decrease of *D* observed for ltPGK-GFP and ltPGK-FRET.

As another control, we studied a protein with a melting temperature between ltPGK and GFP: htPGK-GFP is a mutant of PGK that melts at 44°C *in vitro*, and presumably even higher in cells [24]. Its diffusion coefficient was about 15 µm^2^/s between 22 and 32°C, and increased to 22.5 µm^2^/s at 37°C, without a turnover. Like GFP, the diffusion coefficient of higher-melting htPGK never decreases with increasing temperature.

### PGK-Hsp70 binding in cells

**Figure 6:**
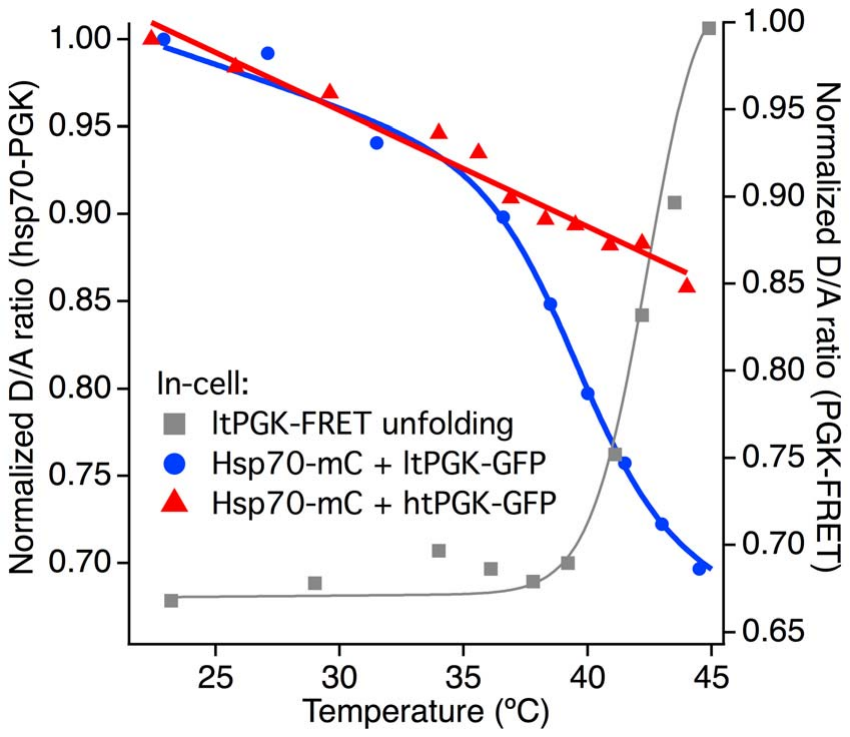
Donor over acceptor (D/A) ratios for protein unfolding and protein-protein interaction. PGK-FRET unfolding (gray) has a midpoint at approximately 42°C for the U2OS cell data shown here, unfolding begins at 37°C. ltPGK-FRET starts interacting with Hsp70-mCherry extensively above 35°C, whereas htPGK-FRET simply continues the room temperature linear trend up to 45°C.

We studied U2OS cells co-expressing the chaperone Hsp70-mCherry and ltGFP-PGK to see if there is binding. Binding is detected by FRET (a decrease of the donor/acceptor  =  D/A ratio) when Hsp binds PGK (see Methods). The cell in Figure 6 shows a binding onset of 35°C and a midpoint of ∼39°C, below the in-cell PGK unfolding transition midpoint. A control experiment conducted with htGFP-PGK shows no significant binding at temperatures below 45°C. A similar experiment conducted with mCherry in place of hsp70-mCherry also shows no significant binding (see Figure S6 in File S1).

## Discussion

Unlike GFP, the diffusion coefficients of ltPGK-GFP and ltPGK-FRET do not increase towards an upper limit when the temperature is raised. Instead, they begin to decrease at 37°C, the highest temperature where reliable measurements could be made over ≈200 s in U2OS cells. We assign this decrease to unfolding of the proteins, which is ≈50% complete between 37 and 39°C. Our simple model, which includes an increased Stokes radius of the unfolded protein, mimics this trend slightly better than a model without unfolding (dotted vs. solid fit curves in Figure 5), but it fails to account quantitatively for the rise and subsequent decrease of *D*.

A possible explanation is that the unfolded proteins do not just have a larger Stokes radius, but that they also ‘stick’ more to other cytoplasmic constituents, thus slowing down diffusion even more than an increased radius alone. Many such interactions, both specific and non-specific, are possible inside the cell. To investigate the plausibility of this idea, we looked at one specific case: PGK is a known client of the heat shock chaperone Hsp70, whose variants are expressed either latently or upregulated upon stress in mammalian cells [30], [34]. The results are summarized in Figure 6. Hsp70-mCherry begins to bind ltPGK-GFP above 35°C in cells, causing the donor/acceptor fluorescence ratio (*D*/*A*) to decrease. PGK that is bound to latent heat shock chaperones as it unfolds could diffuse significantly more slowly than predicted by just its increased Stokes radius in the unfolded state. It will be interesting to see in the future whether binding thermodynamics and kinetics of Hsp70 and its co-chaperone Hsp40 to client proteins differs among aqueous solution, crowders, and live cells, to see how such interactions create some of the ‘stickiness’ that slows down translational diffusion of unfolded proteins in the cell.

The unfolded protein clearly diffuses more slowly than the folded protein, whether due to its larger size, ‘sticking’, or both. A related question raised in the literature is how much crowding or ‘quinary’ interactions (weak association among cytoplasmic constituents) are responsible for retarding translational diffusion of folded proteins in cells [35]. We find that our test proteins fall at most a factor of 2 below our average fitted mass scaling of *γ* = 0.42. GFP and ltPGK-GFP are well fitted by that scaling (Figure 5). At low temperatures, where all our test proteins are folded, crowding and cytoplasmic ‘stickiness’ are thus not a great hindrance for GFP and PGK diffusion. Our data does not support a postulated loose association of folded ltPGK-GFP with other cytoplasmic constituents [35], beyond similar associations formed by GFP.

That is not to say that protein translational diffusion cannot be significantly retarded inside cells at long times and over long distances. Like others [9], we detect anomalous diffusion with *α*<1 in cells, with a range from 0.55–0.9 and an average of 0.64 for the cell shown in Figures 2–4. Based on the goodness of fit in the last column of table 1, anomalous diffusion and spatially heterogeneous diffusion coefficients make similar contributions to the goodness of fit, so both effects seem to play a similar role in deviations of cytosolic diffusion from a single normal diffusion coefficient.

The temperature dependence of the GFP diffusion coefficient is slightly larger than that of water viscosity: it doubles from 22 to 36°C, whereas water viscosity decreases by only 25% over the same temperature range. Thus diffusion of GFP (and below 32°C, also of the two ltPGK variants) speeds up faster than expected from Stokes-Einstein scaling in aqueous solution [36]. One possible explanation is that the protein or the cytoplasmic matrix or both become more flexible at higher temperature, so proteins diffuse through the matrix more easily than expected from the Stokes-Einstein relation. If this is the case, one would expect diffusion coefficients (in the absence of protein unfolding) to increase towards the aqueous solution value as the temperature is raised, but not to exceed that upper limit. Our data is consistent with that expectation.

ltPGK-FRET diffuses somewhat more slowly than suggested by eq. (2) and the thick blue line fit in Figure 5. This could be due to quinary interactions, or due to shape. It is known that the mCherry label on ltPGK-FRET, although engineered not to tetramerize like the wild-type fluorescent protein [37], [38], nonetheless is prone to associating with itself and other proteins. Loose association with other constituents of the cytoplasm could explain ltPGK-FRET's smaller-than-predicted diffusion coefficient at low temperature in Figure 5. As a caveat to this interpretation, the scaling relation in eq. (2) was developed for compact globules, not for multiple tethered globules. An alternative explanation for the slow diffusion of ltPGK-FRET would be that multiple tethered globules diffuse more slowly than a compact globule of same mass in a crowded environment. It would be interesting to see systematic Brownian dynamics simulations comparing such cases in the presence of crowding and non-crowding liquids. It is also possible that hydrodynamic effects play a role in the difference between one large and two small tethered diffusors. To the best of our knowledge, no Brownian dynamics or hydrodynamic simulations currently exist in the literature, but they would help distinguish the ‘sticky mCherry’ and ‘tethered globules diffuse more slowly than a single globule of same mass’ scenarios.

## Supporting Information

File S1 **Supporting Information.** File S1 contains additional simulation details, experimental methods and Figures S1–S6.
(PDF)

Movie S1 **GFP bleaching.** The movie of GFP photobleaching in the cell by FLIP discussed in the main text. The movie is generated by taking the snapshots generated every 10 s during the FLIP measurement and speeding the playback rate to 5 frames per second. The cell shape is selected by thresholding and pixels below the threshold (outside of the cell) are set to 0 for all snapshots. As described in the text, the nucleus of the cell is also selected and pixels inside the nucleus are excluded from simulation and set to 0 for the purpose of illustration. The raw data is obtained as a greyscale image and displayed green since all measured fluorescence is from the GFP. No other modifications or corrections are applied to the data.
(AVI)
