# Supplementary material for: Coupled Protein Diffusion and Folding in the Cell
Source: PLoS One. 2014 Dec 1;9(12):e113040. doi: 10.1371/journal.pone.0113040 (PMC4249841; doi:10.1371/journal.pone.0113040)
Supplement: File S1 — Supporting Information. File S1 contains additional simulation details, experimental methods and Figures S1–S6. (PDF) [file pone.0113040.s001.pdf]

Supplementary Information

Coupled protein diffusion and folding in the cell

Minghao Guo[a], Hannah Gelman[a] and Martin Gruebele[a,b]

[a]Department of Physics, University of Illinois, Urbana, IL 61801, USA

[b]Department of Chemistry, and Center for Biophysics and Computational Biology, University of Illinois, Urbana, IL 61801, USA

The SI discusses additional details of the methods used for data collection and analysis, as well as providing additional examples of data and figures illustrating the analysis. All references to the numerical methods can be found in the Methods section in the main text.

Samples Figure S1 shows the thermodynamic melting curve of ltPGK-GFP as a function of temperature, measured by tryptophan fluorescence using a Cary Eclipse fluorescence spectrophotometer (Varian). PGK conformation was monitored by calculating the spectral center of mass of the tryptophan emission at temperatures from 15 °C to 45 °C. For modeling of in-cell diffusion, the stability was shifted 2.5 °C upward, as described in the main text.

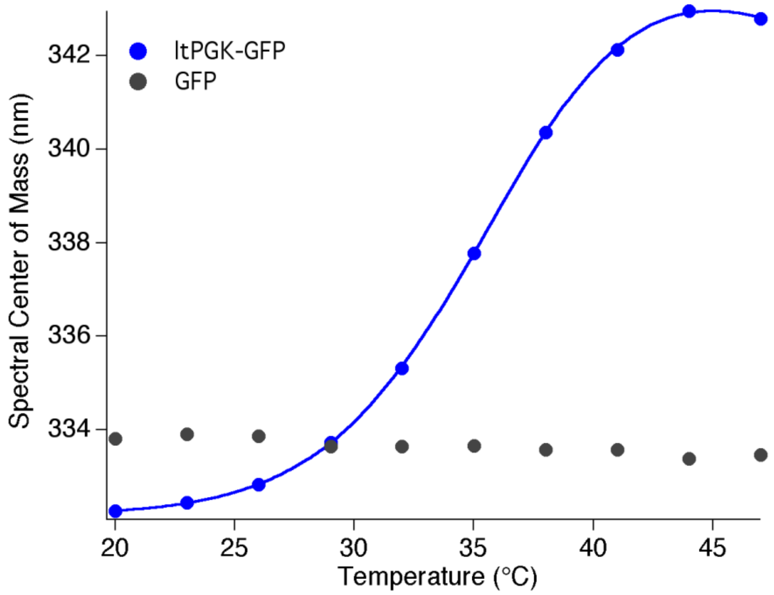

Figure S1. ltPGK-GFP stability, as measured by tryptophan fluorescence. ltPGK-GFP (blue) has a melting temperature of 37 °C (calculated as described in the main text). GFP alone (grey) shows no significant peak shift in the temperature range studied here.

Live cell FLIP and FRAP Cellular diffusion measurements were performed using a Zeiss epi-fluorescence microscope. A 440 nm blue laser is focused onto the cell with a focal spot power of 5 mW with and size of 4 μm in diameter. The laser is combined by a beam splitter with a 470 nm excitation LED for epifluorescent imaging of GFP. In the FLIP measurements, the sample slide was illuminated alternatively by the bleaching laser and the imaging LED. The laser is on

for most of the measurement to bleach molecules that diffuse through the laser spot. Every 10 seconds the laser is turned off and the LED turned on for 0.5 seconds and a snapshot is taken of the GFP intensity distribution throughout the cell (Figure 1B). The cytoplasm is almost totally dark after 20 snapshots (190 seconds of bleaching). The temperature of the sample slide was controlled by a resistive heater and PID controller within 0.1 °C.

The U2OS human bone cancer cell line was used for the live cell diffusion studies. U2OS cells were transfected with a plasmid encoding the protein of interest using Lipofectamine. Transfection in the presence of Lipofectamine was allowed to proceed for 6 hours. Cells were imaged 15-20 hours after the end of transfection. All diffusion experiments were conducted using a GFP tag as both the target of photobleaching and the probe for imaging. GFP is the smallest and most stable construct in this study, it shows no signs of unfolding at temperatures ≥ 65 °C. The "lt"="low temperature" mutant of PGK contains 3 mutations from the wild-type sequence: Y122W/W308F/W333F. ltPGK-FRET has GFP at the N-terminus of PGK and mCherry at the C-terminus. Its unfolding can be detected by FRET *in vitro* and in-cell, yielding $u(T) \approx K_{eq}/(1+K_{eq})$, where $K_{eq}=\exp[-\Delta G/RT]$. $\Delta G=\delta g_T(T-T_m)$ is the two-state unfolding free energy, and the parameters δg_T and T_m were fitted as in refs. (23, 31) in Figure 6. $u(T)$ is shifted *ca.* 2.5 °C more stable in-cell than *in* vitro. Although ltPGK-FRET is not a two-state folder, the approximation is good near T_m. We measured the stability of the lt mutant with a GFP tag at the N-terminus (ltPGK-GFP) by tryptophan fluorescence *in vitro* (see Figure S1) and corrected it by the same *in vitro*-in cell difference measured as measured directly for ltPGK-FRET. The stable mutant htPGK contains Y122W and P111T mutation from wild-type. GFP and htPGK-GFP do not unfold significantly in the experimental temperature range reported here. mCherry is not photobleached and the fluorescence images in the red channel excited by amber light do not change during the experiment on ltPGK-FRET upon excitation by yellow light. This control shows that protein diffusion is free and independent of photobleached GFP.

FRAP measurements were performed with the same setup and cell line. Bleaching at the laser spot was carried out for 100 ms. Immediately after bleaching, a video of fluorescence recovery at the bleaching spot was recorded under LED illumination for 10 seconds at 1000 frames per second (fps). A snapshot taken prior to the application of the bleaching laser pulse was used for reference. The relative intensity change compared to the initial values was fitted to a Gaussian function. The width of the Gaussian function spreads linearly with time, which is used to determine the diffusion coefficient.

The fitted diffusion coefficient (see below and Materials and Methods) was calculated at 4 temperatures (see Table 2 for summary). To demonstrate that the measured diffusion is not affected by the concentration of target proteins, in Figure S2 we plot the diffusion rate with respect to initial fluorescence intensity, where the initial fluorescence intensity (before bleaching) is used as a convenient rough measure of the concentration of target proteins in each cell. We see no clear correlation between the intensity and the diffusion coefficient, demonstrating that interactions between labeled proteins are unlikely to be responsible for differences in diffusion from cell-to-cell.

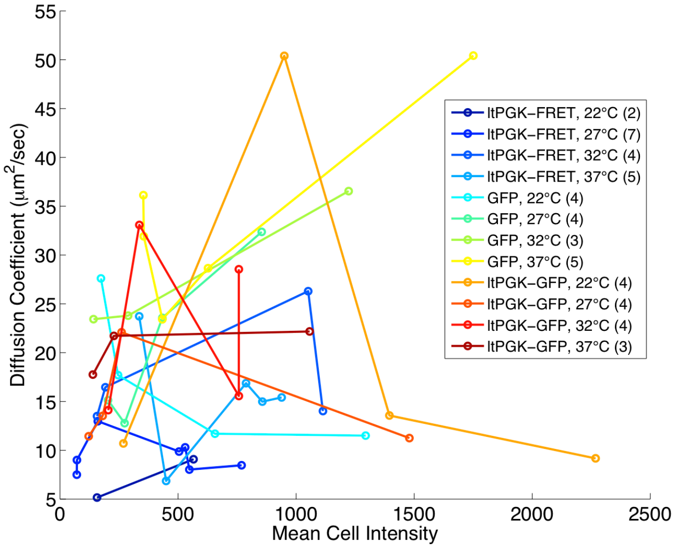

Figure S2. Diffusion coefficient with respect to fluorescence intensity. Each trace represents the distribution of diffusion coefficient and intensity measured for each target protein-temperature pair (summarized in table 2). The number of samples in each pairing is indicated in paranthesis.

Simulation of protein diffusion in cells In the 2-D simulations of diffusion and photobleaching, molecules are allowed to diffuse in a grid area corresponding to the shape of the imaged cell. Grid size is Δx=0.85 μm, corresponding to the pixel resolution of the recorded image, and simulations range 50-100 μm in width, depending on cell size. For 2-D simulations the experimental data is normalized by the initial pixel intensities assuming initial concentration of molecules in the model is uniform. The nucleus is excluded from the diffusion accessible region. Fluorophores in the nucleus remain unbleached throughout the FLIP experiment and no leakage of nuclear fluorescence is observed up to 15 minutes after the conclusion of the experiment. This evidence suggests that there is no significant exchange of target molecules across the nuclear envelope over the course of the experiment.

The simulated laser beam is cylindrically shaped, with Gaussian intensity in the cell plane, σ=2.5 μm. The number of protein molecules photobleached per unit time is proportional to the Gaussian shaped photobleaching rate $B(x,t)=B \cdot A(t) \cdot \exp[-(x^2+y^2)/\sigma^2]$ and molecule concentration $n(x,t)$. $A(t)$=0 (bleaching laser off) or 1 (bleaching laser on) as plotted in Figure 1B. The diffusion of protein molecules follows Fick's law with a concentration sink, representing the bleaching of labeled molecules:

$$\frac{\partial n(x,y,t)}{\partial t} = D\nabla^2 n(x,y,t) - B(x,y,t)n(x,y,t) \quad \text{(S1)}$$

The numerical method to compute the intensity $n_k^{i,j} = n(x_i, y_j, t_k)$, where x_i, y_j are the two spatial dimension and t_k is the discretized time, is:

$$n_{k+1}^{i,j} = n_k^{i,j} + (t_{k+1} - t_k)(D\delta^2 n_k^{i,j} - B_k^{i,j} n_k^{i,j}) \quad \text{(S2)}$$

where $\delta^2 n_k^{i,j} = \frac{1}{\Delta x^2}(n_k^{i+1,j} + n_k^{i-1,j} + n_k^{i,j-1} - 4n_k^{i,j})$. For anomalous diffusion, the analogous fractional diffusion equation

$$\frac{\partial n}{\partial t} = \frac{\partial^{1-\alpha}}{\partial t^{1-\alpha}} D\nabla^2 n - Bn \qquad \text{(S3)}$$

gives the nonlinear power law of mean squared displacement in time $<x^2>\sim t^\alpha$. The numerical method for fractional differential equation is

$$n_{k+1}^{i,j} = n_k^{i,j} + (t_{k+1}^\alpha - t_k^\alpha) D\delta^2 n_k^{i,j} - (t_{k+1} - t_k) B_k^{i,j} n_k^{i,j} \qquad \text{(S4)}$$

The simulated diffusion and bleaching dynamics are computed in the same spatial and temporal range as the experiment. The bleaching rate *B*, diffusion coefficient *D*, and anomalous factor *α* are the parameters adjusted in the least squares fitting to improve the correspondence between the simulated *n*(*x,y,t*) and the experimentally measured spatial distribution of intensities. To improve the reliability of the fitting, the pixels used for fitting are selected using a higher threshold than that used to calculate the cell shape. Parameters for pixels below the higher threshold are determined based on their interaction with the fitted pixels, not by direct comparison with the experimentally measured bleaching (see Figure S5A).

In multi-domain analysis, different values of *D*(*x,y*) and *α*(*x,y*) are used for each domain. The values of *D* and *α* are in the simulation are smoothed using a 10-pixel box filter to avoid sudden changes at the domain boundaries. The following equation was used for the numerical computation of multi-domain anomalous diffusion.

$$n_{k+1}^{i,j} = n_k^{i,j} + \delta_x\left(\left(t_{k+1}^{\alpha^{i,j}} - t_k^{\alpha^{i,j}}\right) D^{i,j}\delta_x n_k^{i,j}\right) + \delta_y\left(\left(t_{k+1}^{\alpha^{i,j}} - t_k^{\alpha^{i,j}}\right) D^{i,j}\delta_y n_k^{i,j}\right) + \Delta t B^{i,j} n_k^{i,j} \qquad \text{(S5)}$$

We also performed the simulation in 3-D for more accurate results. In the 3-D model, the cell depth is proportional to the initial fluorescence intensity without normalization, under the assumption that the concentration of the fluorescence protein in the cell in constant. The nucleus is centered in the cell in the z direction, with accessible space above and beneath it. It is excluded from the diffusion accessible region. The thickness of the nucleus is estimated based on the reduced intensity in that area of the cell and the final fluorescence remaining in the nucleus after the experiment is completed. As above, this assumes that no diffusion of labeled molecules takes place between the nucleus and the cytoplasm over the course of the experiment.

Clustering and partitioning of the cell The fluorescence intensity decay of each pixel in the experiment is first compared with the homogeneous simulation fit (*D*=constant). Pixels with similar dynamics are grouped together based on the residual between the experimental and simulated intensities. Residuals are calculated for all pixels within the cell, including those below the high threshold used for parameter fitting (see Figure S5BC). Each pixel is described by a 19-D vector, representing the differences in fluorescence intensities between experimental and simulated values in 19 snapshots from 10 to 190 s (the first snapshot at 0 s is trivial and excluded). All vectors are grouped into ~10 clusters by the k-means algorithm, ignoring the spatial distribution of the pixels. Then the neighboring clusters are combined and smoothed into the final domains by running the k-means algorithm again. With our signal-to-noise ratio, we found that 3-4 domains accounted for the data within measurement uncertainty.

Visualization of diffusion-photobleaching dynamics The fluorescence intensity decay depends on the location in the cell. A delayed fluorescence intensity decay occurs at pixels

further away from the photobleaching spot. This phase can be cancelled by subtracting the simulation values from the experimental measured values, called residuals, represented in the form of a 19 dimensional vector for the 19 time points sampled in the decay. The anomalous diffusion always results in fast initial intensity decay compared to normal diffusion, but slower decay later. It behaves differently at different locations in the cell due to heterogeneity. Figure S3 shows the intensity decay at two pixels in the cell. If we separate the whole 190 second process sampled by 19 time points into two parts (10-90 s and 100-190 s), calculate the norm of the deviations in these two parts, the 19 residuals can be mapped onto two axes as shown in Figure S3(B). The points closer to the origin represent the pixels in the cell that were accurately calculated in simulation. The points away from origin are the pixels in abnormal local environments with different diffusion behaviors.

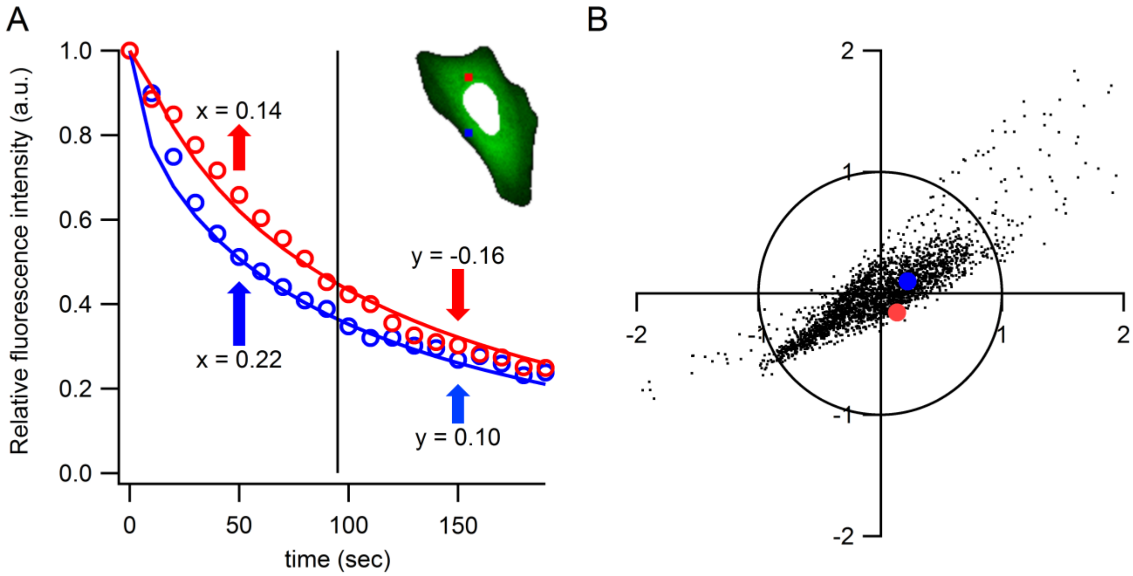

Figure S3. The 2D visualization of the diffusion-photobleaching dynamics. (A) The comparison of fluorescence intensity decay in two selected pixels in the cell shown in inset. The norm of residuals in the first ten and last ten time steps are calculated. (B) All the pixels are plots in a 2D plain based on the x and y values calculated in (A). The red and blue dots represent the two pixels shown in (A).

We calculated the diffusion-photobleaching dynamics with different anomalous diffusion factors α (Figure S4). It is shown that if we use a larger α value (closer to normal diffusion), the fluorescence intensity decays slower than the experimental measured values. A smaller α results in faster initial decay. Only the optimal α value of 0.64 can correctly recover the real anomalous diffusion case (Figure S3(B)).

k-means partitioning of the cell The diffusion-photobleaching dynamics of the cell is calculated with the model of a single diffusion coefficient and anomalous factor. The 19 dimensional deviations of all the pixels are mapped in 2D shown in Figure S3(B) and colored coded as specified in Figure S5(A). We applied the k-means algorithm to group all of the 19-D vectors into 9 clusters shown in Figure S5(B). Larger numbers of clusters could help better determine the local fluctuation of the diffusion behavior in the cell. But some of the clusters are too small and are mixed with neighboring clusters, which could result in unacceptably large uncertainty. Further, using many domains increases the computational cost of the simulation exponentially. To overcome these difficulties, small and neighboring clusters are grouped

together and the edges between clusters are smoothed to form the final 3 domains shown in Figure S5(C) and used for analysis in the main text.

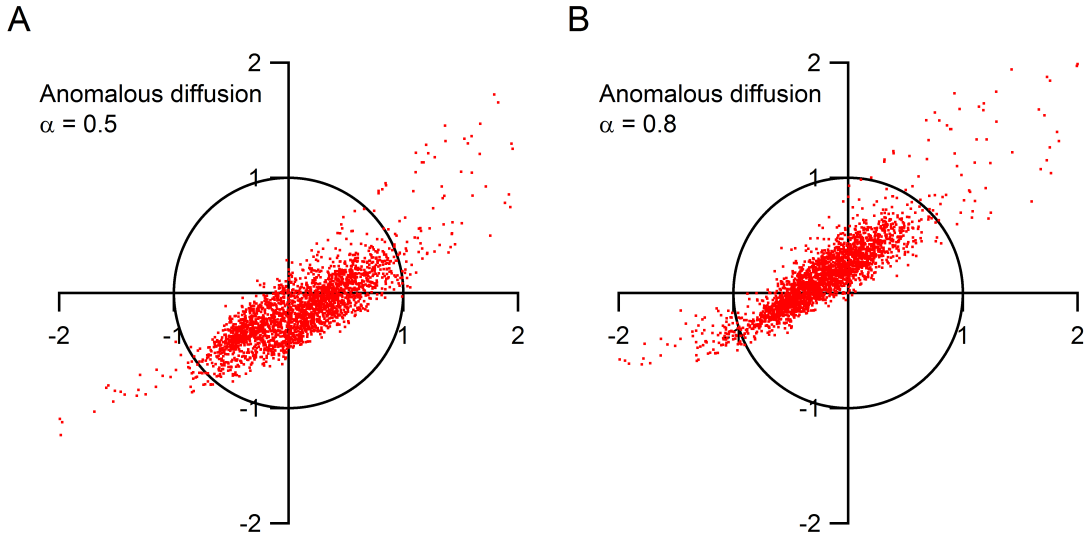

Figure S4. Examples of the effects of anomalous factor α. The diffusion-photobleaching dynamics with smaller α = 0.5 and larger α = 0.8 are shown in (A) and (B). They deviate clearly from the origin.

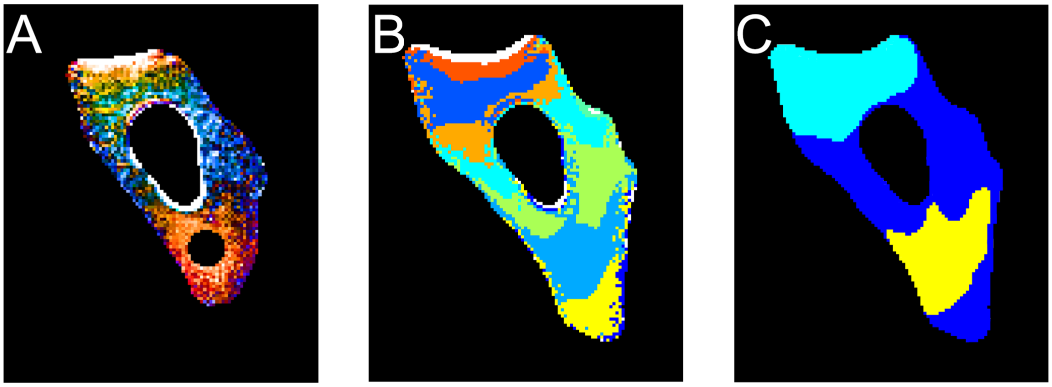

Figure S5. k-means partition of the cell (A) The color-coded deviation of experiment and simulation values in the diffusion-photobleaching dynamics. (B) k-means clustering algorithm was applied to group the pixels with similar dynamics. (C) Small and neighboring clusters are grouped together form the final 3 domains with smoothed edges. The cell shape shown in A is smaller than that in B,C and the main text because it shows the higher intensity pixels used for parameter fitting, while clustering and simulation was done over the whole cell shape.

PGK-Hsp70 binding in the cell Hsp-70 binding experiments were conducted using the same ltPGK-GFP and htPGK-GFP constructs as in the diffusion experiments. A fluorescent hsp70 fusion protein was created by cloning the sequence for the human, cytoplasmic, heat-inducible hsp70 (hsp72) with a C-terminal mCherry tag into the pDream 2.1/MCS vector (Genscript Corp., Piscataway, NY). The construct also includes N-terminal FLAG and hexahistidine tags. C-

terminal fluorescent protein fusions of Hsp-70 have been shown to maintain chaperone activity and to co-express and co-localize with native Hsp70 under heat shock conditions (31). Binding experiments were conducted in cells by co-transfecting U2OS cells with Hsp70 and PGK plasmids as described in the main text. Cells were imaged for binding experiments 12-15 hours after the beginning of transfection. Binding experiments were conducted as described in (24). Briefly, red and green fluorescence from mCherry and GFP was separated into two channels by a dichroic mirror and imaged side-by-side on a CMOS camera sensor. Cells were imaged under steady illumination at temperatures from 20 °C – 45 °C to create a temperature titration curve.

Binding between hsp70-mCherry and GFP-PGK was detected *via* FRET from GFP to mCherry. FRET was quantified as in (24): the ratio of average donor to acceptor fluorescence (D/A) was calculated for each cell at each temperature. As the interaction between the Hsp70-mCherry and GFP-PGK increases, the FRET efficiency between the two fluorescent tags increases and D/A decreases. The change in the ratio D/A with respect to temperature was fitted by a two-state model identical to the one described in the FLIP and FRAP Methods section.

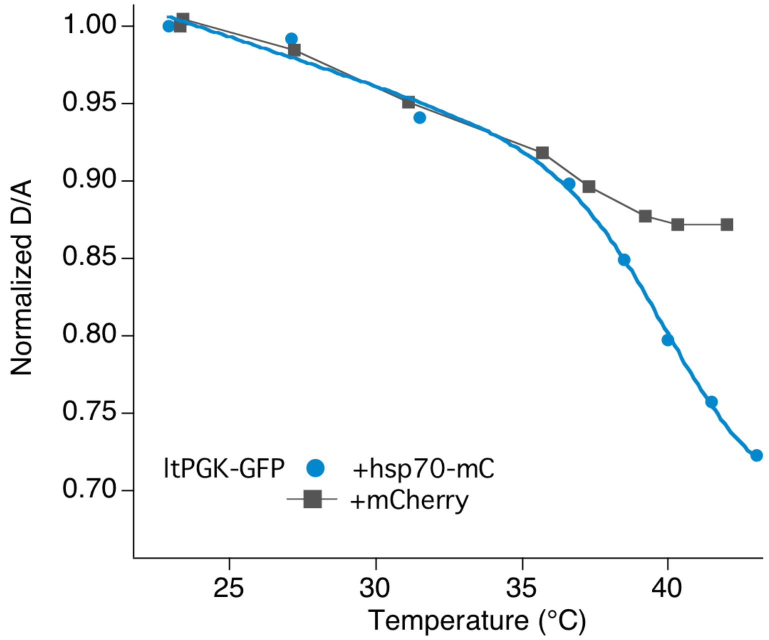

Figure S6. A control experiment monitors the interaction between mCherry and ltPGK-GFP (grey) in U2OS cells. A comparison with the hsp70-mCherry binding (blue) shows that no significant binding takes place between the mCherry and ltPGK-GFP near the melting temperature of the ltPGK-GFP.
